# Risk factors for self-reported diabetes among Bhutanese adults: A nationally representative survey data analysis

**DOI:** 10.1371/journal.pone.0206034

**Published:** 2018-11-15

**Authors:** Kinley Wangdi, Tshering Jamtsho

**Affiliations:** 1 Phuentsholing General Hospital, Phuentsholing, Bhutan; 2 Department of Global Health, Research School of Population Health, College of Health and Medicine, The Australian National University, Canberra, Australia; 3 School of Demography, ANU College of Arts & Social Sciences, The Australian National University, Canberra, Australia; Nagoya University, JAPAN

## Abstract

**Background:**

Bhutan, a small land-locked country in the eastern Himalayas has been undergoing an epidemiological and nutritional transition in the last two decades. The objective of this paper was to determine the prevalence and explore the risk factors of self-reported diabetes among Bhutanese adults.

**Methods:**

We conducted a secondary data analysis among adults 18 years and older Bhutanese using the data from the National Health Survey 2012 (NHS, 2012) of Bhutan. The self-reported information on risk factors was obtained using standard protocols of the WHO STEPwise approach to Surveillance. The outcome of interest was self-reported diabetes on medication. Adjusted and unadjusted logistic regression analyses were performed to identify the risk factor of diabetes.

**Results:**

A total of 31,066 participants aged 18 years and older were included for the analysis. The prevalence of self-reported hypertension was 1.8% (491). Risk factors for diabetes were: age groups: 35–44 years adjusted odds ratio (AOR) = 2.82 (95% CI, 1.07, 7.41), 45–54 years AOR = 6.02 (95% CI, 2.29, 15.83), 55–64 year AOR = 15.7 (95% CI 5.93, 41.55) and >65 years AOR = 19.60 (95% CI, 6.93, 55.71); high school and diploma/certificate education AOR = 2.57, (95% CI 1.62, 4.07) and AOR = 3.92 (95% CI 1.70, 9.07); and urban dwellers AOR = 2.37, (95% 1.58, 3.57); hypertension AOR = 3.3, (95% CI 2.47, 4.41); and fruit servings of 1–3 per week AOR = 1.63 (95% CI 1.15, 2.31).

**Conclusion:**

The number of Bhutanese adults with diabetes and co-morbidities associated with it is likely to increase with the ageing of the population, nutrition transition, and high rural-urban migration in the near future. This calls for an urgent need to implement strategies to prevent diabetes in the Bhutanese population targeting risk factors including healthy lifestyle with increased physical activities and reduced smoking. At the same time treating other chronic morbidities including hypertension.

## Introduction

Diabetes Mellitus (DM) is a major public health problem globally with an estimated 422 million adults living with DM in 2014 [1]. Worldwide DM prevalence has increased over the last 30 years and now ranks in the top four high priority non-communicable diseases (NCDs) of the World Health Organisation (WHO) [2]. By 2035, DM is projected to reach 592 million cases [3–5] mainly in middle and low-income countries [6]. DM requires lifelong care and treatment which incur massive health care costs. The costs associated with diabetes health care expenditure was at least USD$ 376 billion worldwide in 2010 and will increase to USD$ 490 billion by 2030 [7, 8].

The aetiology of diabetes is multifactorial and includes modifiable and non-modifiable risk factors. The non-modifiable risk factors include genetics and age [9], while modifiable risk factors are obesity [10], sedentary lifestyle [11], an unhealthy diet that contains a high level of sugar [12–14] and saturated fats [15], and smoking [16–19]. The modifiable risk factors are associated with rising living standards, rural-urban migration, and lifestyle changes [20, 21]. Diabetes is a health challenge fuelled by rapidly ageing populations and lifestyle risk factors, in particular, smoking and obesity [3, 22, 23]. Whilst advances in treatment have extended life expectancy, diabetes and its associated complications and comorbidities have been found to result in increased long-term physical disability [24, 25].

Bhutan, with a population of 757,042 [26, 27] has been undergoing an epidemiological and nutritional transition in the last two decades [28]. This transition is characterised by rising disposable income levels, a shift from traditional high-carbohydrate, low-fat diets towards diets lower in carbohydrates and higher in saturated fat, sugar and salt, and lower levels of physical activity [29]. Further, demographics are changing rapidly as young people in particular move to towns for work, education and entertainment [30–32]. Despite this societal transformation, no prior systematically analysed in-depth study representing the whole national prevalence exists on diabetes and its associated risk factors. Therefore, the primary objective of this paper is to determine the prevalence and explore the risk factors for diabetes among the adult Bhutanese. Findings from this study will provide a better understanding of the risk factors of diabetes. This knowledge can be used to guide policy changes and initiate a national preventive programme targeting these risk factors.

## Methods

### Study population

This study utilized secondary, a nationally representative data from the National Health Survey (NHS 2012) conducted in all the 20 districts of Bhutan in 2012. The survey samples were estimated using the census sample frame adopted for the first Population and Housing Census of Bhutan (PHCB, 2005) in 2005. The survey samples were appropriately derived and designed to produce statistically reliable estimates of most indicators at the national and 20 districts level, further aggregated by rural and urban categorization. The study participants were adult older than 18 years and the outcome variable of interest was self-reported diabetes under medication.

### Risk factor assessment

The variables were all self-reported and followed the WHO STEPwise approach to Surveillance of non-communicable diseases (STEP) survey guidelines. The outcome variable of interest was self-reported diabetes mellitus (DM) defined as elevated blood sugar requiring medication to lower the blood sugar [33]. One fruit serving included either: (a) ½ cup of chopped, cooked or canned fruit; or (b) 1 medium-sized piece of fruit such as banana, apple, orange; or (c) ½ cup of fruit juice (not artificially flavoured). One standard vegetable serving was: (a) 1 cup of raw green leafy vegetable such as spinach, salad greens, etc. or (b) ½ cup of other vegetables, cooked or chopped, such as carrots, pumpkin, corn, beans, onion, etc., but excluding tubers such as potatoes. A “standard drink” was the amount of ethanol contained in standard glasses of beer, wine, fortified wine such as sherry, and spirits and usually between 8–13 grams. Vigorous activity was defined as an activity that results in increased breathing or heart rate similar to carrying or lifting heavy loads, digging or construction work for at least 10 minutes.

### Statistical analysis

Estimation procedures included sampling weights to adjust for the complex sample design. All statistical analyses were weighted and performed using the appended sample weights of respective survey data-set (individual module) as to provide nationally representative estimates. The individual module data-set comprised appended sample weights that were adjusted, calculated and normalised in accordance with the survey sample weights calculation technique. Sample weights were thus available for the public use for further analysis of survey data. The analysis was performed using the SVY module for complex samples of the statistical package STATA version 15 (Stata Corporation, College Station, TX, USA). Univariate and multivariate logistic regression models for risk factors of diabetes were built using backward elimination to identify significant covariates. A value of p ≤ 0.05 was considered statistically significant for any observed differences.

## Results

### Socio-demographic characteristics of study population

A total of 31,066 participants aged 18–75 years were included for the analysis. The men made up 46.1% (14,335) of the samples, and the median age of the study subject was 37 years (range 18–75). A fourth of the study participants were in the age group of 25–34 years (8,060) and more than 75.0% (23,420) participants were from rural areas. Half (15,666) of the participants had no formal education, 20.2% (6,260) completed high school and less than 1% (275) had completed a diploma or certificate level education. The majority of participants were married 74.1% (22,985). Farmers, unskilled and clerical workers and service and sales workers made up 66.1% (10,061) and 20.7% (3,155) of participants. The self-reported hypertension was 17.4% (5,408). Alcohol intake and smoking were reported in 47.3% (14,681) and 4.2% (1,322) of the study population.

A high proportion of study participants 55.4% (16,744) did not consume any fruit, however, only a small percentage reported not consuming enough vegetables, 3.0% (900). Vegetables were consumed throughout the week with varying durations; 41.0% (12,420) consumed vegetables 6–7 days a week, 30.3% (9,206) consumed vegetables 4–5 days a week, and 25.7% (7,788) consumed vegetables 1–3 days a week. Physical (vigorous) activities were reported by 47.4% (14,725) of the study participants (**Table 1**).

**Table 1:** Sociodemographic characteristics of study population and diabetes participants in Bhutan.

Variables	Total (%) N = 31,065	Diabetes (%) N = 491
**Gender**		
Women	16,730 (53.9)	249 (50.7)
Men	14,335 (46.1)	242 (49.3)
**Age group (years)**		
18–24	6,083 (19.6)	15 (3.1)
25–34	8,060 (26.0)	34 (6.8)
35–44	6,172 (19.9)	75 (15.2)
45–54	5,100 (16.4)	104 (21.3)
55–64	3,543 (11.4)	162 (33.1)
≥65	2,107 (7.8)	100 (20.5)
**Urban-rural**		
Rural	23,420 (75.4)	186 (37.9)
Urban	7,645 (24.6)	304 (62.1)
**Education**		
No formal education	15,666 (50.6)	281 (57.4)
Non-formal education	2,560 (8.3)	25 (5.0)
Primary school	3,820 (12.3)	57 (11.6)
High school	6,260 (20.2)	74 (15.1)
Diploma/Certificate	275 (0.9)	11 (2.3)
University	1,501 (4.8)	16 (3.3)
Monastic education	899 (2.9)	26 (5.0)
**Marital status**		
Single	5,223 (16.8)	13 (2.7)
Married	22,985 (74.1)	400 (81.6)
Divorced/Separated	1,299 (4.2)	28 (5.6)
Widowed	1,531 (4,9)	50 (10.1)
**Occupation**		
Clerical/farmer/unskilled	10,061 (66.1)	127 (58.9)
Army	540 (3.5)	7 (3.3)
Manager and professionals	1,339 (8.8)	25(11.5)
Service and sales worker	3,155 (20.7)	51 (23.6)
Monks	134 (0.9)	6 (5.6)
**Hypertension**		
No	25,646 (82.6)	218 (44.5)
Yes	5,408 (17.4)	272 (55.5)
**Alcohol intake**		
No	16,378 (62.7)	266 (54.3)
Yes	14,681 (47.3)	224 (45.7)
**Current smoker**		
No	29,741 (95.8)	469 (95.7)
Yes	1,322 (4.2)	21 (4.3)
**Ever smoked**		
No	26,067 (83.9)	378 (77.1)
Yes	4,999 (16.1)	112 (22.9)
**Fruit serving per week**		
No	16,744 (55.4)	207 (42.9)
1–3 days	8,352 (27.6)	165 (34.2)
4–5 days	2,916 (9.6)	61 (12.8)
6–7 days	2,239 (7.4)	49 (10.1)
**Vegetable servings per week**		
No	900 (3.0)	14 (2.8)
1–3 days	7,789 (25.7)	103 (21.4)
4–5 days	9,206 (30.3)	145 (30.1)
6–7 days	12,420 (41.0)	220 (45.7)
**Vigorous physical activities**		
No	16,334 (52.6)	320 (65.3)
Yes	14,725 (47.4)	170 (34.7)

Source: **National Health Survey 2012**

### Socio-demographic characteristics of diabetic patients

The prevalence of diabetes in this study was 1.8% (491) and there were equal men and women 49.3% (242) to 50.7% (249). More than one fifth (101) of diabetic patients were older than 65 years. Sixty-two percent (304) of diabetic patients were from urban areas. More than half (281) of diabetic patients did not have formal education, and 3.3% (16) were university educated. Eighty-two percent (400) of diabetic individuals were married and only 2.7% (13) were single. Of 419 diabetic patients, occupation was recorded for 215 participants. The commonest occupation of diabetic patients were farmers (clerical and unskilled), followed by service and sales workers at 58.9% (127) and 23.6% (51) respectively. Forty-six percent (224) had concurrent hypertension, and more than half consumed alcohol (54.7%, 224), 4.3% (21) were current smokers and 22.9% (112) who had ever smoked. Forty-three percent (207) did consume fruits in a typical week, while 12.6% (62), 30% (147) and 10.1% (15) consumed fruits in 1–3, 4–5 and 6–7 days respectively. Only 34.7% (170) engaged in vigorous physical activities (**Table 1**).

### Smoking and alcohol use stratified by sex

There was a difference between men and women in smoking and alcohol. More than 7% (1,044) of men were current smokers as compared only 1.7% (277) of women. Similarly, men were more than thrice 25.1% (3,592) likely to have ever smoked than women 8.4% (1,407). However, the difference in alcohol use between the men and women were much closer, 58.5% (8,386) of men ever consumed alcohol while it was 37.6% (6,295) for women. Men tend to be younger for both current smokers (mean age 32.5 years) and ever smoked (mean age 40 years) as compared to women. However, there was no age difference between men (mean age 41.2 years) and women (mean age 41.1 years) who ever consumed alcohol (**Table 2**).

**Table 2:** Smoking and alcohol use stratified by sex in Bhutan.

Characteristics		Sex			
		Men		Women	
		Number (%)	Mean age*	Number (%)	Mean age*
Current smoker					
	No	13,290 (92.7)	40.3	16,451 (98.3)	38.3
	Yes	1,044 (7.3)	32.5	277 (1.7)	40.4
Ever smoked					
	No	10,743 (74.9)	39.6	15,324 (91.6)	37.8
	Yes	3,592 (25.1)	40.0	1,407 (8.4)	44.2
Ever consumed alcohol					
	No	5,944 (41.5)	37.6	10,435 (62.4)	36.3
	Yes	8,386 (58.5)	41.2	6,295 (37.6)	41.1

* years

Source: **National Health Survey 2012**

### Factors associated with diabetes

Older than 35 years, being a single and widow, high school educated, having hypertension, living in urban areas, ever smoked and engaging in a vigorous physical activity were each independently associated with hypertension in univariate analysis.

After adjusting for other variables, participants in age groups of 35–44 years, 45–54, 55–65 and above 65 years were approximately adjusted odds ratio (AOR) = 2.82 (95% CI, 1.07, 7.42), AOR = 6.03 (95% CI, 2.29, 15.85), AOR = 15.74 (95% CI 5.95, 41.63) and AOR = 19.82 (95% CI, 6.99, 56.21) more at risk of diabetes compared to participants aged 18–24 years. High school and diploma educated AOR = 2.58, (95% CI 1.63, 4.09) and AOR = 3.02 (95% CI 1.70, 9.06) were at higher risk of diabetes in comparison with no education group. Hypertension was a major risk factor for diabetes; participants with hypertension were 3.3 times more likely to have diabetes as compared to those who do not report having hypertension (AOR = 3.30, 95% CI 2.47, 4.41). Urban dwellers were 2.37 times more at risk of diabetes to rural inhabitants (AOR = 2.37, 95% 1.58, 3.57). Interestingly fruit servings of 1–3 per week were associated with diabetes risk as compared to those who do not consume fruits (AOR = 1.63, 95% CI 1.15, 2.31) (**Table 3**).

**Table 3:** Univariate and multivariate logistic regression analysis with risk factors for diabetes in Bhutan.

Variable	Unadjusted Correlates			Adjusted Correlates		
	OR^†^	95% CI*	p-value	OR^†^	95% CI*	p-value
**Sex**						
Men	1			1		
Women	0.91	0.76, 1.09	0.288	0.85	0.57, 1.26	0.416
**Age group**						
18–24	1			1		
25–34	1.66	0.91, 3.06	0.1	0.79	0.29, 2.15	0.65
35–44	5.00	2.88, 8.70	<0.001	2.82	1.07, 7.42	0.035*
45–54	8.71	5.08, 14.95	<0.001	6.03	2.29, 15.85	<0.001*
55–64	21.47	12.66, 36.42	<0.001	15.74	5.95, 41.63	<0.001*
>65	24.31	14.12, 41.85	<0.001	19.82	6.99, 56.21	<0.001*
**Marital status**						
Single	1			1		
Married	7.40	4.26, 12.83	<0.001	1.42	0.60, 3.37	0.425
Separated^‡^	9.19	4.75, 17.80	<0.001	1.77	0.61, 5.14	0.219
Widow	16.14	8.75, 29.76	<0.001	0.72	0.22, 2.35	0.583
**Education**						
No education	1			1		
Primary	0.74	0.56, 0.99	0.42	1.39	0.90, 2.15	0.142
High	0.53	0.41, 0.69	<0.001	2.58	1.63, 4.09	<0.001*
University or equivalent	0.48	0.29, 0.79	0.004	1.63	0.76, 3.50	0.213
Diploma/ certificate	1.90	1.04, 3.48	0.038	3.92	1.70, 9.06	0.001*
Monk	1.44	0.95, 2.16	0.082	1.34	0.68, 2.65	0.403
NFE	0.49	0.32, 0.74	0.001	0.48	0.13, 1.78	0.270
**Occupation**						
Clerical/farmer /unskilled	1			1		
Army	0.89	0.41, 1.90	0.76	0.62	0.27, 1.42	0.255
Manager and professionals	1.21	0.79, 1.88	0.382	0.62	0.35, 1.12	0.111
Service and sales worker	1.13	0.81, 1.57	0.465	0.85	0.57, 1.27	0.436
Monks	3.36	1.42, 7.94	0.006	1.72	0.64, 4.62	0.283
**Urban-rural**						
Rural	1			1		
Urban	1.64	1.38, 1.98	<0.001	2.37	1.58, 3.57	<0.001*
**Hypertension**						
No	1			1		
Yes	6.04	5.04, 7.24	<0.001	3.30	2.47, 4.41	<0.001*
**Current Smoker**						
No	1					
Yes	0.99	0.65, 1.55	1.0	-	-	-
**Ever smoked**						
No	1			1		
Yes	1.52	1.23, 1.88	<0.001	1.27	0.92, 1.75	0.154
**Ever consumed alcohol**						
No	1					
Yes	0.96	0.80, 1.14	0.618	-	-	-
**Fruit servings days per week**						
0	1			1		
1–3	1.48	1.20, 1.82	<0.001	1.63	1.15, 2.31	0.006*
4–5	1.56	1.17, 2.09	0.002	1.56	0.98, 2.50	0.062
6–7	1.62	1.18, 2.22	0.003	1.38	0.82, 2.35	0.23
**Vigorous physical activity**						
No	1					
Yes	0.62	0.51, 0.74	<0.001	0.95	0.69, 1.30	0.731

^†^OR- odds ratio

^*^CI-confidence interval

^‡^Separated include those divorced and living separately

Source: **National Health Survey 2012**

## Discussion

This is the first epidemiological study on the prevalence of diabetes and its risk factors in the adult Bhutanese. The data for this study was obtained from the National Health Survey 2012. The national prevalence of diabetes was estimated at 1.8%, which is lower to 2010 global diabetes prevalence of 6.4% [34]. The risk of diabetes is likely to increase as Bhutan undergoes an epidemiological, a nutritional and a demographic transition. Further, increased life expectancy will predispose a higher proportion of the population to the risk of diabetes. In the light of these changes, it is important to initiate a preventive programme targeting these modifiable diabetes risk factors. The significant modifiable risk factors for diabetes in this study were: high school and diploma or certificate education level, hypertension and urban dwellers.

Age is an important non-modifiable risk factor for diabetes [9, 35, 36] and this highlights the risk of pre-diabetic respondents progressing to develop diabetes if appropriate measures are not undertaken. As people live longer in Bhutan due to improvement in the healthcare, the cost of managing diabetes and its complication is likely to increase the medical expenditure. Diabetes-related morbidity and mortality have been increasing each year in Bhutan [37, 38]. This calls for public health interventions through education on a regular screening of blood sugar of older people using both pharmacological and non-pharmacological control of any elevated blood sugar to avert complications associated with it.

Urbanities were at the risk of suffering from diabetes in this study which is similar to the findings from the neighbouring countries [39–42]. There are a number of reasons such as the rapid urbanization, industrialization, and modernization leading to less physical activity at work, travelling to and from work, and at leisure, resulting in a more sedentary lifestyle [43]. Studies have shown that urban dwellers are more likely to consume food containing higher levels of sugar including sugar-sweetened beverages than rural residents [12, 44–48]. Other study showed that urban poor were at higher risk than rural people [49], suggesting that those that are well educated can choose to adopt a healthy lifestyle, the poor have fewer food choices and more limited access to nutritional education [50]. The diabetic burden in Bhutan is expected to increase with increased rural-urban migration in the coming years [30–32].

Participants with education levels of high school and diploma holders were at a higher risk of diabetes as compared to those without education as reported in other studies [35]. However, other literature reported an increased risk with lesser education [51]. The risk could be associated with an unhealthy diet as compared to the physical activity because the occupation was not a risk factor in this study.

Diabetes and hypertension are closely linked morbidities [21, 52–54], with obesity being a common risk factor for hypertension and diabetes. In Bhutan, it is estimated that around 40% of those aged 15 and above are overweight or obese [55]. The coexistence of hypertension with diabetes is associated with a six-fold increase in the risk of cardiovascular events compared to the general population [56]. Therefore, it is important to initiate aggressive sustained intervention in people with diabetes to control blood pressure and lower body weight [57–59].

Physical activity offered protection against diabetes in univariate analysis consistent with other published studies [16, 60, 61]. Regular physical activities including walking, cycling, jogging, and swimming should be advocated because it may prevent or delay type 2 diabetes development [11, 60, 62]. In addition, the benefits of a physical activity extend beyond protection against diabetes [63]. Regular physical exercise can improve lipid profile, decrease body weight, lower blood pressure, and reduce cardiovascular disease risk [64–66]. This reduction in cardiovascular disease risk in individuals with diabetes is very important because they are twice at risk of experiencing serious cardiovascular disease events and are two to four times more likely to die from the complications of cardiovascular events compared to the general population [67, 68].

The main strength of the study is that this is the first nationally representative study on the risk of diabetes and risk factors. However, this study is subjected to a number of limitations. The study is subjected to probable recall bias. The behavioural risk factors in this study (i.e. fruit and consumption, tobacco use, alcohol use, and physical activity) were self-reported which could have led to under or overestimates of the actual levels of risk factors. In communities where certain behaviours are discouraged, there may be under-reporting of these behaviours (e.g. alcohol and tobacco consumption), especially among females. Additionally, the applied method of self-reporting diabetes status is neither able to provide data in relation to the level of diabetes's awareness (thus the evaluated prevalence of diabetes may be underestimated) nor can be as accurate as clinical examination. This study, given its cross-sectional approach, cannot establish causal relations, but rather can only generate hypotheses that could be evaluated by future prospective randomized trials.

## Conclusion

The number of Bhutanese adults with diabetes and co-morbidities associated with it is likely to increase with the ageing of the population, nutrition transition, and high rural-urban migration in the near future. This calls for an urgent need to implement strategies to prevent diabetes in the Bhutanese population targeting risk factors including healthy lifestyle with increased physical activities, and treating co-morbidities including hypertension. In addition, there is also a need for epidemiological studies to monitor the trends of diabetes and related risk factors, as well as outcome, both fatal and non-fatal.

## References

[1] WHO. Global report on diabetes. World Health Organization, Geneva, Switzerland 2016 10.2337/db15-0956

[2] WHO. Global Status Report on noncommunicable diseases 2010. Geneva, Switzerland; 2010.

[3] International Diabetes Federation. IDF Diabetes Atlas 7th edition IDF; 2016 [1 August ]. Available from: http://www.diabetesatlas.org

[4] Guariguata L, Whiting DR, Hambleton I, Beagley J, Linnenkamp U, Shaw JE. Global estimates of diabetes prevalence for 2013 and projections for 2035. Diabetes research and clinical practice. 2014;103(2):137–49. Epub 2014/03/19. 10.1016/j.diabres.2013.11.002 . 24630390 10.1016/j.diabres.2013.11.002

[5] Wild S, Roglic G, Green A, Sicree R, King H. Global prevalence of diabetes: estimates for the year 2000 and projections for 2030. Diabetes care. 2004;27(5):1047–53. Epub 2004/04/28. . 15111519 10.2337/diacare.27.5.1047

[6] WHO. Diabetes Fact sheet: World Health Orginization; 2016 [cited 2017 March 22,]. Available from: http://www.who.int/mediacentre/factsheets/fs312/en/.

[7] Zhang P, Zhang X, Brown J, Vistisen D, Sicree R, Shaw J, et al Global healthcare expenditure on diabetes for 2010 and 2030. Diabetes research and clinical practice. 2010;87(3):293–301. Epub 2010/02/23. 10.1016/j.diabres.2010.01.026 . 20171754 10.1016/j.diabres.2010.01.026

[8] Seuring T, Archangelidi O, Suhrcke M. The Economic Costs of Type 2 Diabetes: A Global Systematic Review. PharmacoEconomics. 2015;33(8):811–31. Epub 2015/03/20. 10.1007/s40273-015-0268-9 ; PubMed Central PMCID: PMCPMC4519633. 25787932 10.1007/s40273-015-0268-9 PMC4519633

[9] Shrivastava SR, Ghorpade AG. High prevalence of type 2 diabetes melitus and its risk factors among the rural population of Pondicherry, South India. Journal of research in health sciences. 2014;14(4):258–63. Epub 2014/12/17. . 25503279

[10] Misra A, Khurana L. Obesity-related non-communicable diseases: South Asians vs White Caucasians. International journal of obesity (2005). 2011;35(2):167–87. Epub 2010/07/21. 10.1038/ijo.2010.135 . 20644557 10.1038/ijo.2010.135

[11] Baum K, Votteler T, Schiab J. Efficiency of vibration exercise for glycemic control in type 2 diabetes patients. International journal of medical sciences. 2007;4(3):159–63. Epub 2007/06/08. ; PubMed Central PMCID: PMCPMC1885552. 17554399 10.7150/ijms.4.159 PMC1885552

[12] Lofvenborg JE, Andersson T, Carlsson PO, Dorkhan M, Groop L, Martinell M, et al Sweetened beverage intake and risk of latent autoimmune diabetes in adults (LADA) and type 2 diabetes. European journal of endocrinology. 2016;175(6):605–14. Epub 2016/12/08. 10.1530/EJE-16-0376 . 27926472 10.1530/EJE-16-0376

[13] Malik VS, Popkin BM, Bray GA, Despres JP, Willett WC, Hu FB. Sugar-sweetened beverages and risk of metabolic syndrome and type 2 diabetes: a meta-analysis. Diabetes care. 2010;33(11):2477–83. Epub 2010/08/10. 10.2337/dc10-1079 ; PubMed Central PMCID: PMCPMC2963518. 20693348 10.2337/dc10-1079 PMC2963518

[14] Imamura F, O'Connor L, Ye Z, Mursu J, Hayashino Y, Bhupathiraju SN, et al Consumption of sugar sweetened beverages, artificially sweetened beverages, and fruit juice and incidence of type 2 diabetes: systematic review, meta-analysis, and estimation of population attributable fraction. British journal of sports medicine. 2016;50(8):496–504. Epub 2016/04/06. 10.1136/bjsports-2016-h3576rep ; PubMed Central PMCID: PMCPMC4853528. 27044603 10.1136/bjsports-2016-h3576rep PMC4853528

[15] van Dam RM, Willett WC, Rimm EB, Stampfer MJ, Hu FB. Dietary fat and meat intake in relation to risk of type 2 diabetes in men. Diabetes care. 2002;25(3):417–24. Epub 2002/03/05. . 11874924 10.2337/diacare.25.3.417

[16] Shi L, Shu XO, Li H, Cai H, Liu Q, Zheng W, et al Physical activity, smoking, and alcohol consumption in association with incidence of type 2 diabetes among middle-aged and elderly Chinese men. PloS one. 2013;8(11):e77919 Epub 2013/11/14. 10.1371/journal.pone.0077919 ; PubMed Central PMCID: PMCPmc3817165. 24223743 10.1371/journal.pone.0077919 PMC3817165

[17] Chang SA. Smoking and Type 2 Diabetes Mellitus. Diabetes Metab J. 2012;36(6):399–403. 10.4093/dmj.2012.36.6.399 PubMed PMID: PMC3530709. 23275932 10.4093/dmj.2012.36.6.399 PMC3530709

[18] Radzeviciene L, Ostrauskas R. Smoking habits and the risk of type 2 diabetes: a case-control study. Diabetes & metabolism. 2009;35(3):192–7. Epub 2009/03/10. 10.1016/j.diabet.2008.11.001 . 19269874 10.1016/j.diabet.2008.11.001

[19] Willi C, Bodenmann P, Ghali WA, Faris PD, Cornuz J. Active smoking and the risk of type 2 diabetes: a systematic review and meta-analysis. Jama. 2007;298(22):2654–64. Epub 2007/12/13. 10.1001/jama.298.22.2654 . 18073361 10.1001/jama.298.22.2654

[20] Jayawardena R, Ranasinghe P, Byrne NM, Soares MJ, Katulanda P, Hills AP. Prevalence and trends of the diabetes epidemic in South Asia: a systematic review and meta-analysis. BMC public health. 2012;12:380 Epub 2012/05/29. 10.1186/1471-2458-12-380 ; PubMed Central PMCID: PMCPmc3447674. 22630043 10.1186/1471-2458-12-380 PMC3447674

[21] Geldsetzer P, Manne-Goehler J, Theilmann M, Davies JI, Awasthi A, Vollmer S, et al Diabetes and Hypertension in India: A Nationally Representative Study of 1.3 Million Adults. JAMA internal medicine. 2018;178(3):363–72. Epub 2018/01/31. 10.1001/jamainternmed.2017.8094 ; PubMed Central PMCID: PMCPMC5885928. 29379964 10.1001/jamainternmed.2017.8094 PMC5885928

[22] Hu FB, Satija A, Manson JE. Curbing the diabetes pandemic: The need for global policy solutions. Jama. 2015;313(23):2319–20. 10.1001/jama.2015.5287 25996138 10.1001/jama.2015.5287 PMC5291074

[23] Stewart Williams J, Ling R, Searles AM, Doran CM, Byles J. Identification of higher hospital costs and more frequent admissions among mid-aged Australian women who self-report diabetes mellitus. Maturitas. 2016;90:58–63. 10.1016/j.maturitas.2016.04.008 27282795 10.1016/j.maturitas.2016.04.008

[24] Huo L, Shaw JE, Wong E, Harding JL, Peeters A, Magliano DJ. Burden of diabetes in Australia: life expectancy and disability-free life expectancy in adults with diabetes. Diabetologia. 2016;59(7):1437–45. 10.1007/s00125-016-3948-x 27075450 10.1007/s00125-016-3948-x

[25] Wong E, Woodward M, Stevenson C, Backholer K, Sarink D, Peeters A. Prevalence of disability in Australian elderly: Impact of trends in obesity and diabetes. Preventive medicine. 2016;82:105–10. Epub 2015/11/21. 10.1016/j.ypmed.2015.11.003 . 26586499 10.1016/j.ypmed.2015.11.003

[26] Office of the Census Commissioner. Results of Population & Housing, Census of Bhutan 2005. 2006.

[27] NSB. Dzongkhag Population Projections 2006–2015. In: National Stastics Bureau RGoB, editor. Thimphu, Bhutan2008.

[28] B.M. P, S. H, S. K. THE NUTRITIONAL TRANSITION AND DIET-RELATED CHRONIC DISEASES IN ASIA: IMPLICATIONS FOR PREVENTION. In: Food Consumption and Nutrition Division IFPRI, editor. Washington, D.C.2001.

[29] Popkin BM, Adair LS, Ng SW. NOW AND THEN: The Global Nutrition Transition: The Pandemic of Obesity in Developing Countries. Nutrition Reviews. 2012;70(1):3–21. 10.1111/j.1753-4887.2011.00456.x PubMed PMID: PMC3257829. 22221213 10.1111/j.1753-4887.2011.00456.x PMC3257829

[30] Gosai MA, Sulewski L. Urban attraction: Bhutanese internal rural–urban migration. Asian Geographer. 2014;31(1):1–16. 10.1080/10225706.2013.790830

[31] Khandu T. Causes of Rural Urban Migration in Bhutan: Cornell University; 2006.

[32] Chand R, editor Rural Urban Migration in Bhutan: a Study in Local and Regional Responses and Marginalization. IGU Regional Conference; 2014.

[33] MoH. National National Health Survey 2012. Ministry of Health, Thimphu, Bhutan: 2012.

[34] Shaw JE, Sicree RA, Zimmet PZ. Global estimates of the prevalence of diabetes for 2010 and 2030. Diabetes research and clinical practice. 2010;87(1):4–14. Epub 2009/11/10. 10.1016/j.diabres.2009.10.007 . 19896746 10.1016/j.diabres.2009.10.007

[35] Akter S, Rahman MM, Abe SK, Sultana P. Nationwide survey of prevalence and risk factors for diabetes and prediabetes in Bangladeshi adults. Diabetes care. 2014;37(1):e9–e10. Epub 2013/12/21. 10.2337/dc13-1647 . 24356610 10.2337/dc13-1647

[36] Ono K, Limbu YR, Rai SK, Kurokawa M, Yanagida J, Rai G, et al The prevalence of type 2 diabetes mellitus and impaired fasting glucose in semi-urban population of Nepal. Nepal Medical College journal: NMCJ. 2007;9(3):154–6. Epub 2007/12/21. . 18092429

[37] MoH. Annual Health Bulletin. In: Minstry of Health B, editor. Thimphu, Bhutan2016.

[38] MoH. Annual Health Bulletin. In: Ministry of Health B, editor. Thimphu, Bhutan2015.

[39] Ramachandran A, Mary S, Yamuna A, Murugesan N, Snehalatha C. High prevalence of diabetes and cardiovascular risk factors associated with urbanization in India. Diabetes care. 2008;31(5):893–8. Epub 2008/03/04. 10.2337/dc07-1207 . 18310309 10.2337/dc07-1207

[40] Akter S, Rahman MM, Abe SK, Sultana P. Prevalence of diabetes and prediabetes and their risk factors among Bangladeshi adults: a nationwide survey. Bulletin of the World Health Organization. 2014;92(3):204–13, 13a. Epub 2014/04/05. 10.2471/BLT.13.128371 ; PubMed Central PMCID: PMCPmc3949596. 24700980 10.2471/BLT.13.128371 PMC3949596

[41] Dong Y, Gao W, Nan H, Yu H, Li F, Duan W, et al Prevalence of Type 2 diabetes in urban and rural Chinese populations in Qingdao, China. Diabetic medicine: a journal of the British Diabetic Association. 2005;22(10):1427–33. Epub 2005/09/24. 10.1111/j.1464-5491.2005.01658.x . 16176207 10.1111/j.1464-5491.2005.01658.x

[42] Saquib N, Khanam MA, Saquib J, Anand S, Chertow GM, Barry M, et al High prevalence of type 2 diabetes among the urban middle class in Bangladesh. BMC public health. 2013;13:1032 Epub 2013/11/01. 10.1186/1471-2458-13-1032 ; PubMed Central PMCID: PMCPmc3924340. 24172217 10.1186/1471-2458-13-1032 PMC3924340

[43] Hu FB. Sedentary lifestyle and risk of obesity and type 2 diabetes. Lipids. 2003;38(2):103–8. Epub 2003/05/08. . 12733740 10.1007/s11745-003-1038-4

[44] McCloskey ML, Tarazona-Meza CE, Jones-Smith JC, Miele CH, Gilman RH, Bernabe-Ortiz A, et al Disparities in dietary intake and physical activity patterns across the urbanization divide in the Peruvian Andes. The international journal of behavioral nutrition and physical activity. 2017;14(1):90 Epub 2017/07/12. 10.1186/s12966-017-0545-4 ; PubMed Central PMCID: PMCPmc5504673. 28693514 10.1186/s12966-017-0545-4 PMC5504673

[45] Bansal D, Satija A, Khandpur N, Bowen L, Kinra S, Prabhakaran D, et al Effects of migration on food consumption patterns in a sample of Indian factory workers and their families. Public health nutrition. 2010;13(12):1982–9. Epub 2010/05/29. 10.1017/S1368980010001254 . 20507672 10.1017/S1368980010001254

[46] Bowen L, Ebrahim S, De Stavola B, Ness A, Kinra S, Bharathi AV, et al Dietary intake and rural-urban migration in India: a cross-sectional study. PloS one. 2011;6(6):e14822 Epub 2011/07/07. 10.1371/journal.pone.0014822 ; PubMed Central PMCID: PMCPmc3120774. 21731604 10.1371/journal.pone.0014822 PMC3120774

[47] Chinnakali P, Upadhyay RP, Shokeen D, Singh K, Kaur M, Singh AK, et al Prevalence of household-level food insecurity and its determinants in an urban resettlement colony in north India. Journal of health, population, and nutrition. 2014;32(2):227–36. Epub 2014/08/01. ; PubMed Central PMCID: PMCPmc4216959. 25076660 PMC4216959

[48] Satija A, Hu FB, Bowen L, Bharathi AV, Vaz M, Prabhakaran D, et al Dietary patterns in India and their association with obesity and central obesity. Public health nutrition. 2015;18(16):3031–41. Epub 2015/02/24. 10.1017/S1368980015000312 ; PubMed Central PMCID: PMCPmc4831640. 25697609 10.1017/S1368980015000312 PMC4831640

[49] Misra A, Pandey RM, Devi JR, Sharma R, Vikram NK, Khanna N. High prevalence of diabetes, obesity and dyslipidaemia in urban slum population in northern India. International journal of obesity and related metabolic disorders: journal of the International Association for the Study of Obesity. 2001;25(11):1722–9. Epub 2001/12/26. 10.1038/sj.ijo.0801748 . 11753596 10.1038/sj.ijo.0801748

[50] Kearney J. Food consumption trends and drivers. Philosophical transactions of the Royal Society of London Series B, Biological sciences. 2010;365(1554):2793–807. Epub 2010/08/18. 10.1098/rstb.2010.0149 ; PubMed Central PMCID: PMCPmc2935122. 20713385 10.1098/rstb.2010.0149 PMC2935122

[51] Murad MA, Abdulmageed SS, Iftikhar R, Sagga BK. Assessment of the common risk factors associated with type 2 diabetes mellitus in jeddah. International journal of endocrinology. 2014;2014:616145 Epub 2014/12/31. 10.1155/2014/616145 ; PubMed Central PMCID: PMCPmc4165874. 25548563 10.1155/2014/616145 PMC4165874

[52] Williams B. Treating hypertension in patients with diabetes: When to start and how low to go? Jama. 2015;313(6):573–4. 10.1001/jama.2015.89 25668260 10.1001/jama.2015.89

[53] Cheung BMY, Li C. Diabetes and Hypertension: Is There a Common Metabolic Pathway? Current Atherosclerosis Reports. 2012;14(2):160–6. 10.1007/s11883-012-0227-2 PubMed PMID: PMC3314178. 22281657 10.1007/s11883-012-0227-2 PMC3314178

[54] Ferrannini E, Cushman WC. Diabetes and hypertension: the bad companions. Lancet. 380(9841):601–10. 10.1016/S0140-6736(12)60987-8 22883509 10.1016/S0140-6736(12)60987-8

[55] WHO. WHO Global InfoBase (Database). 2009.

[56] Ninomiya T, Kubo M, Doi Y, Yonemoto K, Tanizaki Y, Rahman M, et al Impact of metabolic syndrome on the development of cardiovascular disease in a general Japanese population: the Hisayama study. Stroke. 2007;38(7):2063–9. Epub 2007/05/26. 10.1161/STROKEAHA.106.479642 . 17525396 10.1161/STROKEAHA.106.479642

[57] Whaley-Connell A, Sowers JR. Hypertension management in type 2 diabetes mellitus: recommendations of the Joint National Committee VII. Endocrinology and metabolism clinics of North America. 2005;34(1):63–75. Epub 2005/03/09. 10.1016/j.ecl.2004.11.007 . 15752922 10.1016/j.ecl.2004.11.007

[58] Berlowitz DR, Ash AS, Hickey EC, Glickman M, Friedman R, Kader B. Hypertension management in patients with diabetes: the need for more aggressive therapy. Diabetes care. 2003;26(2):355–9. Epub 2003/01/28. . 12547862 10.2337/diacare.26.2.355

[59] Stamler J, Vaccaro O, Neaton JD, Wentworth D. Diabetes, other risk factors, and 12-yr cardiovascular mortality for men screened in the Multiple Risk Factor Intervention Trial. Diabetes care. 1993;16(2):434–44. Epub 1993/02/01. . 8432214 10.2337/diacare.16.2.434

[60] Colberg SR, Sigal RJ, Yardley JE, Riddell MC, Dunstan DW, Dempsey PC, et al Physical Activity/Exercise and Diabetes: A Position Statement of the American Diabetes Association. Diabetes care. 2016;39(11):2065–79. Epub 2016/12/08. 10.2337/dc16-1728 . 27926890 10.2337/dc16-1728 PMC6908414

[61] Shaw JE, Chisholm DJ. 1: Epidemiology and prevention of type 2 diabetes and the metabolic syndrome. The Medical journal of Australia. 2003;179(7):379–83. Epub 2003/09/25. . 14503906 10.5694/j.1326-5377.2003.tb05677.x

[62] Sigal RJ, Armstrong MJ, Colby P, Kenny GP, Plotnikoff RC, Reichert SM, et al Physical activity and diabetes. Canadian journal of diabetes. 2013;37 Suppl 1:S40–4. Epub 2014/05/16. 10.1016/j.jcjd.2013.01.018 . 24070962 10.1016/j.jcjd.2013.01.018

[63] Hayes C, Kriska A. Role of physical activity in diabetes management and prevention. Journal of the American Dietetic Association. 2008;108(4 Suppl 1):S19–23. Epub 2008/05/28. 10.1016/j.jada.2008.01.016 . 18358249 10.1016/j.jada.2008.01.016

[64] Haskell WL, Lee IM, Pate RR, Powell KE, Blair SN, Franklin BA, et al Physical activity and public health: updated recommendation for adults from the American College of Sports Medicine and the American Heart Association. Medicine and science in sports and exercise. 2007;39(8):1423–34. Epub 2007/09/01. 10.1249/mss.0b013e3180616b27 . 17762377 10.1249/mss.0b013e3180616b27

[65] Haskell WL, Lee IM, Pate RR, Powell KE, Blair SN, Franklin BA, et al Physical activity and public health: updated recommendation for adults from the American College of Sports Medicine and the American Heart Association. Circulation. 2007;116(9):1081–93. Epub 2007/08/03. 10.1161/CIRCULATIONAHA.107.185649 . 17671237 10.1161/CIRCULATIONAHA.107.185649

[66] Sadarangani KP, Hamer M, Mindell JS, Coombs NA, Stamatakis E. Physical activity and risk of all-cause and cardiovascular disease mortality in diabetic adults from Great Britain: pooled analysis of 10 population-based cohorts. Diabetes care. 2014;37(4):1016–23. Epub 2014/03/22. 10.2337/dc13-1816 . 24652727 10.2337/dc13-1816

[67] Buse JB, Ginsberg HN, Bakris GL, Clark NG, Costa F, Eckel R, et al Primary prevention of cardiovascular diseases in people with diabetes mellitus: a scientific statement from the American Heart Association and the American Diabetes Association. Circulation. 2007;115(1):114–26. Epub 2006/12/29. 10.1161/CIRCULATIONAHA.106.179294 . 17192512 10.1161/CIRCULATIONAHA.106.179294

[68] Hu G, Jousilahti P, Barengo NC, Qiao Q, Lakka TA, Tuomilehto J. Physical activity, cardiovascular risk factors, and mortality among Finnish adults with diabetes. Diabetes care. 2005;28(4):799–805. Epub 2005/03/29. . 15793176 10.2337/diacare.28.4.799

